# Protective effect of low dose intra-articular cadmium on inflammation and joint destruction in arthritis

**DOI:** 10.1038/s41598-017-02611-5

**Published:** 2017-05-25

**Authors:** Paola Bonaventura, Guillaume Courbon, Aline Lamboux, Fabien Lavocat, Hubert Marotte, Francis Albarède, Pierre Miossec

**Affiliations:** 1 Department of Immunology and Rheumatology, Immunogenomics and inflammation research Unit EA 4130, University of Lyon, Lyon, 69437 France; 2 Department of Bone and Osteoarticular Biology INSERM U1059 University Jean Monet, University of Lyon, Saint-Etienne, 42100 France; 3 Geology Laboratory – Department of Earth Sciences, Ecole Normale Supérieure de Lyon and CNRS Lyon, Lyon, 69364 France

## Abstract

Synovium hyperplasia characterizes joint diseases, such as rheumatoid arthritis (RA). The cytotoxic effect of low-dose Cadmium (Cd) was tested *in vitro* and *ex vivo* on synoviocytes, the mesenchymal key effector cells of inflammation and proliferation in arthritis. The anti-inflammatory and anti-proliferative effects of Cd were tested *in vivo* by intra-articular injection in the adjuvant induced arthritis rat joints, where the clinical scores and the consequences of arthritis were evaluated. Cell death through apoptosis was highly induced by Cd in inflammatory synoviocytes (80% reduction of cell viability, p < 0.01). TNF plus IL-17 cytokine combination induced a two-fold increase of Cd cell content by enhancing the ZIP-8 importer and the MT-1 homeostasis regulator expression. Addition of Cd reduced IL-6 production in TNF plus IL-17-activated synoviocytes (up to 83%, p < 0.05) and in *ex-vivo* synovium biopsies (up to 94%, p < 0.01). Cd-injection in rat joints improved arthritis, reducing clinical scores (arthritic score reduced from 4 to 2, p < 0.01), inflammatory cell recruitment (up to 50%, p < 0.01) and protecting from bone/cartilage destruction. This proof of concept study is supported by the limited Cd spread in body reservoirs, with low-dose Cd providing a safe risk/benefit ratio, without toxic effects on other cell types and organs.

## Introduction

Synovium hyperplasia is a critical hallmark of inflammatory joint diseases. The production of cytokines from infiltrated immune cells maintains the chronically-inflamed status^1, 2^. IL-17 synergistically with TNF-α induces IL-6 production from synoviocytes^3^, the mesenchymal cells of the synovium, which in response to inflammation develop resistance to apoptosis^4^. The mesenchymal hypothesis of joint inflammation proposes that, after an initial event triggered by T cells, synovitis is maintained by both autocrine and paracrine pathways involving mainly synoviocytes^5^.

Over the past 20 years, the understanding of the immune/inflammatory mechanisms has led to the development of systemic anti-inflammatory therapy^6, 7^. Nevertheless there is a limit to the response to biologic treatments and clinical remission does not necessarily correspond to non-progression of joint damage^8^. Limited progress has been made in the intra-articular therapy^9^, even if there is a long list of arthritis conditions with unmet need where such approach would be useful, ranging from some forms of oligo-articular rheumatoid arthritis (RA) and juvenile idiopathic arthritis (JIA) to pigmented villonodular synovitis (PVNS). Abandoning the use of radioactive synovectomy^10^, no current therapy uses a mesenchymal approach, targeting synoviocytes. Several metals show an interesting potential for the modulation of inflammation and cell viability, with the example of Zinc (Zn) upregulating IL-6 production in synoviocytes^11^. Zinc and cadmium (Cd) are closely associated for their chemical properties and oxidative stress induction mechanisms^12, 13^. Their common cellular transporters are Zrt-Irt-Proteins (ZIPs 1–14) importers, Zn-Transporters (ZnTs 1–10, with ZnT1 as the only membrane exporter) and metallothioneins (MT-1 and -2), controlling metal homeostasis in cells^14^. In addition, Cd can compete with Zn in the Zn-MT complex.

While Zn is necessary for a normal immune response^15, 16^, Cd inhibits the activity of anti-oxidative enzymes, mitochondrial electron transport-chain^17^ and metalloproteins^18^ with deleterious effects on cells. The possible contribution of Cd to RA is still unclear, ranging from its role as contributor to the induction of RA^19^, to its protective effect in a mouse model^20^.

Based on the effects of Cd on cells^21, 22^ and its pro-apoptotic properties^23, 24^, Cd was tested *in vitro*, *ex vivo* and *in vivo* in the context of arthritis. This proof of concept study indicates that Cd has an anti-proliferative and anti-inflammatory effect in the local context of arthritis. Although metals have their own limitations due to toxicity, as seen with the use of cisplatin in cancer^25^, the quantities of Cd used in this study are infinitesimal and such intra-articular approach could have a positive risk/benefit ratio.

## Results

### Low-dose Cd exposure reduces cell viability and decreases IL-6 production by cytokine-exposed synoviocytes

**Figure 1:**
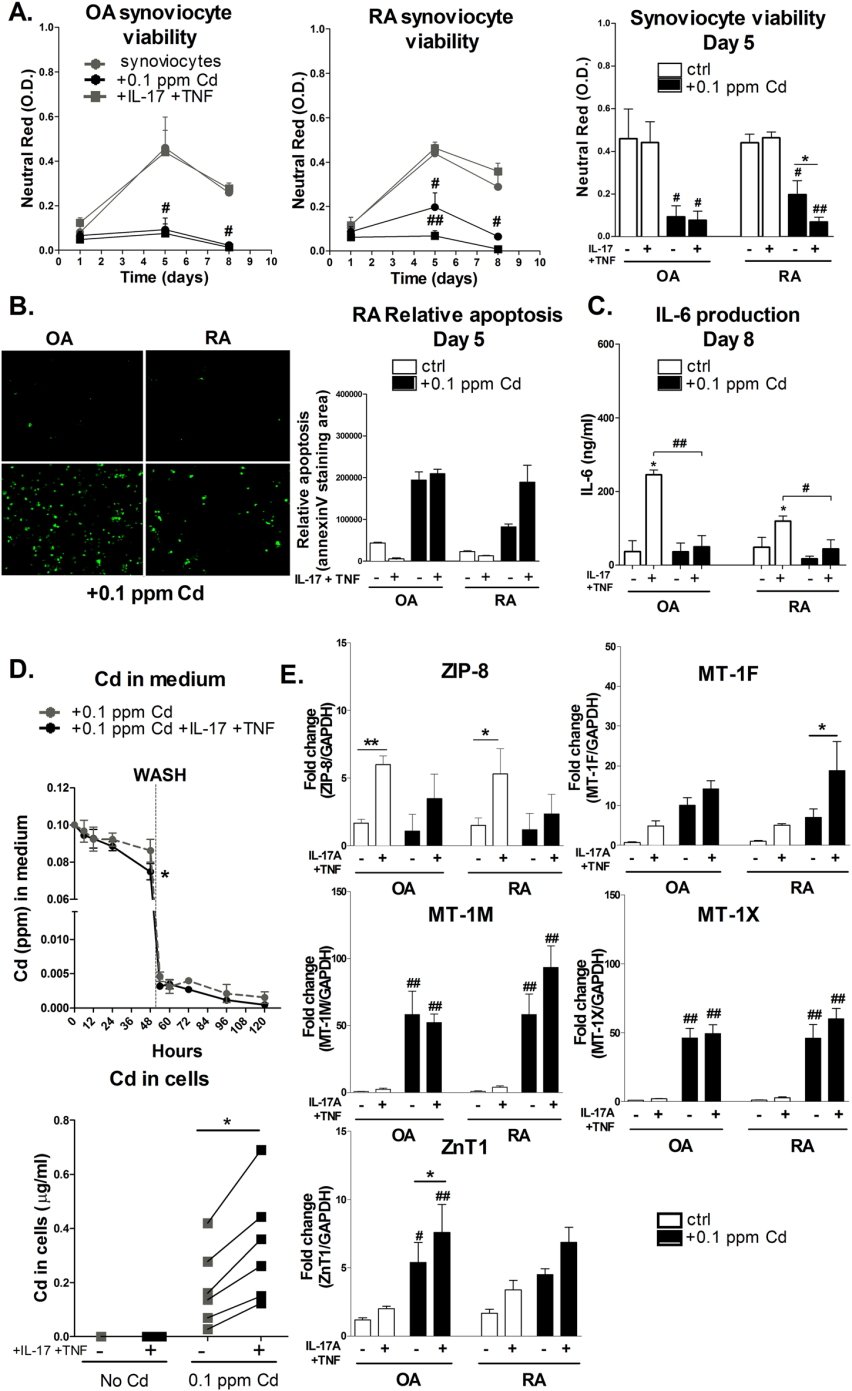
Inflammation drives ZIP-8 and MT-s mediated Cd absorption and accumulation in synoviocytes, inducing apoptosis and reducing IL-6 production. (**A**) Cell viability of OA and RA synoviocyte cultures assessed by neutral red assay at day 1, 5 and 8 after exposure to Cd. (**B**) Annexin V staining of OA and RA synoviocytes exposed or not to Cd for 5 days in the presence or not of IL-17 and TNF. (**C**) IL-6 production of OA and RA synoviocyte cultures assessed by ELISA assay at day 8 after exposure to Cd. (**D**) Cd concentration in medium as function of time and in synoviocyte culture at 120 hours. Gray dashed line: control experiment with no cytokine addition. Black line: Cd plus IL-17 and TNF condition. Vertical dashed line at 48 hours: washing. (**E**) Gene expression of Cd transporters in comparison to GAPDH. Black bars and lines: exposure to Cd. Data are the mean of at least three independent cell lines/biopsies (from three independent patients). Data are represented as mean ± SEM; *shows differences between non-inflammatory and inflammatory conditions, ^#^shows differences due to Cd addition; *^/#^p < 0.05, **^/##^p < 0.01.

The effect of low-dose (0.1 ppm) Cd was tested in an inflammatory context as would be the case in inflammatory joints, by adding or not the inflammatory cytokines IL-17 and TNF-α to synoviocytes from patients with RA and OA, a less inflammatory form of arthritis. For cells not exposed to Cd, the peak of cell viability was at day 5. Both OA and RA Cd-exposed synoviocytes showed an early-stage reduced viability at day 5 (p < 0.05). At day 8, the number of Cd-treated viable cells was less than 10% of the controls, indicating a massive cytotoxic effect. Interestingly, cell death was delayed in RA synoviocytes exposed to Cd-only, compared to both Cd and IL-17/TNF-α combination (Fig. 1A, p < 0.01), but not in OA cells, demonstrating RA-acquired refractoriness to cell death. Annexin-V quantification indicated that Cd enhanced apoptosis in OA synoviocytes, independently from inflammation, while in RA synoviocytes the effect of Cd alone was further enhanced by the IL-17/TNF-α and Cd combination (Fig. 1B). Moreover, RA synoviocytes were less sensitive than OA synoviocytes to IL-17/TNF stimulation for the production of IL-6, probably as a consequence of previous exposure of RA synoviocytes to inflammation at the synovial site. The addition of Cd to the inflammatory condition and the consequent reduction of cell viability was associated with a reduction of IL-6 production by both OA and RA cytokine-treated synoviocytes at day 8 (Fig. 1C). Such reduced inflammation are in line with the apoptotic cell death process.

### ZIP-8 and MT-1 drive Cd accumulation in synoviocytes exposed to the IL-17 and TNF-α combination

To understand how pro-inflammatory cytokines influenced Cd accumulation in synoviocytes, kinetics and transporter expression analysis were performed. Cytokine/Cd combination increased Cd absorption relative to Cd-only exposed synoviocytes (0.07 ± 0.01 ppm *vs*. 0.09 ± 0.01 ppm; p < 0.05, at 48 hours) (Fig. 1D). Even after washing, cytokine-treated synoviocytes kept absorbing residual Cd from medium, showing a net positive bias in favour of Cd absorption, even at small concentrations. Cd intracellular level at the end-point was increased in the Cd plus IL-17/TNF-α-exposed synoviocytes (0.34 ± 0.09 ppm with versus 0.18 ± 0.06 ppm without cytokines; p < 0.05). Moreover, Cd accumulation was not reversible since Cd was not excreted in the medium after washing.

IL-17/TNF-induced-inflammation had a pivotal role in the expression of the Zn/Cd importer ZIP-8 (Fig. 1E; ZIP-8 expression without vs. with cytokines: p < 0.01 in OA and p < 0.05 in RA synoviocytes). Cd addition slightly reduced the cytokine effect. The expression of MT-1M and MT-1X was enhanced by a factor of 40 and 60 by Cd-exposure (p < 0.01) in both cell types. MT-1F expression was only marginally up-regulated by Cd alone, but the presence of cytokines enhanced this effect (Fig. 1E; p < 0.05). The expression of the exporter ZnT1 was significantly enhanced in OA synoviocytes exposed to Cd but to a lesser extent in RA synoviocytes. The net effect was an increased intracellular uptake and storage of Cd by cytokine-treated synoviocytes.

### Low-dose Cd exposure reduces cell viability and decreases IL-6 production in co-cultures and synovial explants

**Figure 2:**
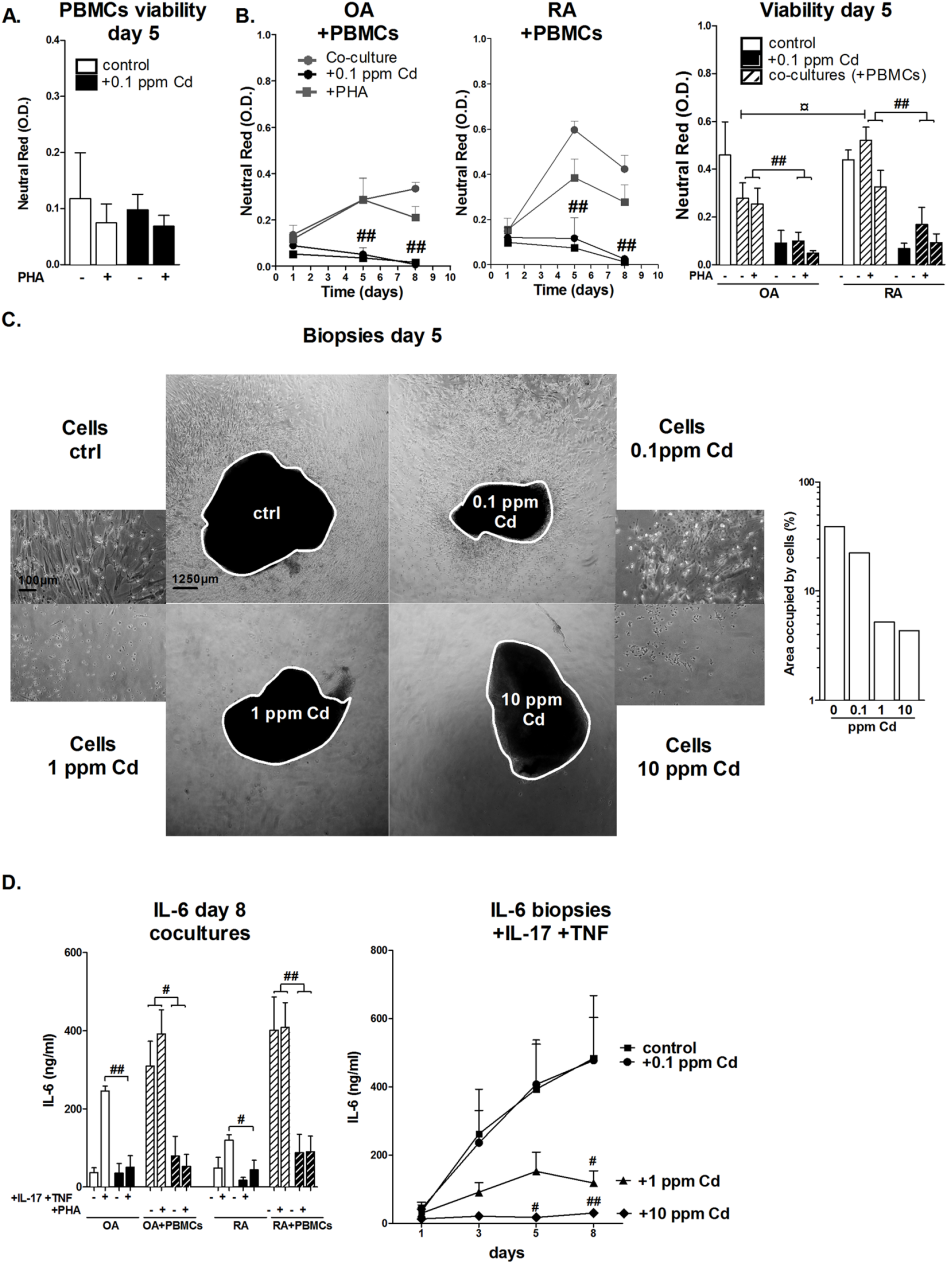
Low-dose Cd addition to RA synoviocyte cultures and biopsies, in inflammatory conditions, reduces cell viability and IL-6 production. (**A**) PBMCs viability assessed by neutral-red assay after exposure to Cd at 0.1 ppm. (**B**) OA and RA synoviocyte viability in co-culture with PBMCs assessed by neutral red assay at day 1, 5 and 8 after exposure to Cd. (**C**) Phase contrast photographs of biopsies (4x) and cells (10x) after exposure to IL-17 and TNF combination in the presence of increasing concentrations of Cd and quantification of the area occupied by the cells. (**D**) IL-6 production measured in supernatants of co-cultures after PHA activation or not and biopsies after exposure to IL-17 and TNF combination, with or without Cd addition at increasing concentrations. Data are the mean of at least three independent cell lines/biopsies (from three independent patients). Data are represented as mean ± SEM; Black lines: Cd exposure. *Indicates differences between non-inflammatory and inflammatory conditions, ^#^differences due to Cd addition only in comparison to control; *^/#^p < 0.05, **^/##^p < 0.01.

Cells in the synovium interact in two major ways: cytokine secretion or direct cell-cell interaction^26^. As already described, PBMCs strongly enhance IL-6 production from synoviocytes only when there is a direct cellular contact^27^. A co-culture model with PBMC and synoviocytes was developed to reproduce the cell interactions in inflamed synovium. PBMC alone exposed to 0.1 ppm of Cd showed no change of viability after 5 days of culture (Fig. 2A), in the presence or not of PHA. When PBMC, activated or not, were co-cultured with synoviocytes, the viability was slightly reduced in comparison to synoviocyte alone, except for RA synoviocytes co-cultured with non-activated PBMCs (Fig. 2B). The addition of Cd induced a strong reduction of cell viability in co-cultures as described above in cytokine-treated isolated synoviocytes (Fig. 2B, p < 0.01). Similarly, IL-6 production was reduced in Cd-treated co-cultures (Fig. 2D, p < 0.01).

To extend this observation to the disease situation, Cd was added to RA synovium biopsies activated with IL-17/TNF-α. Cell proliferation outgrowth from the biopsy to the plate was measured as the area occupied by the cells on the plate, which was reduced by Cd in a dose-dependent manner (Fig. 2C, p < 0.05 for 1 and 10 ppm). In control supernatants, the level of IL-6 increased from day 1 to 8, up to a concentration of 750 ng/ml. Cd at 0.1 ppm did not reduce IL-6 production relative to controls. Higher Cd concentrations strongly reduced IL-6 production at day 8 as low as ~150 ng/ml with Cd used at 1 ppm (p < 0.05) and ~10 ng/ml with Cd used at 10 ppm (p < 0.01) (Fig. 2D). The reduction of IL-6 production was proportional to the amount of Cd, reflecting cell death in synoviocytes.

### Cadmium intra-articular injection controls inflammation-induced joint destruction in the adjuvant induced arthritis Lewis rat model

AIA is an acute inflammatory model of arthritis with joint swelling and periarticular bone loss, particularly at the ankle site^28^.

Cadmium diluted in physiological serum at 0.1, 1 and 10 ppm was injected into the ankles, at the stage of active arthritis (day 14), as it would be the case in the human clinical situation. These concentrations correspond to the final injection of 5, 50 and 500 ng of Cd, respectively. The control group received the physiological solution only.

**Figure 3:**
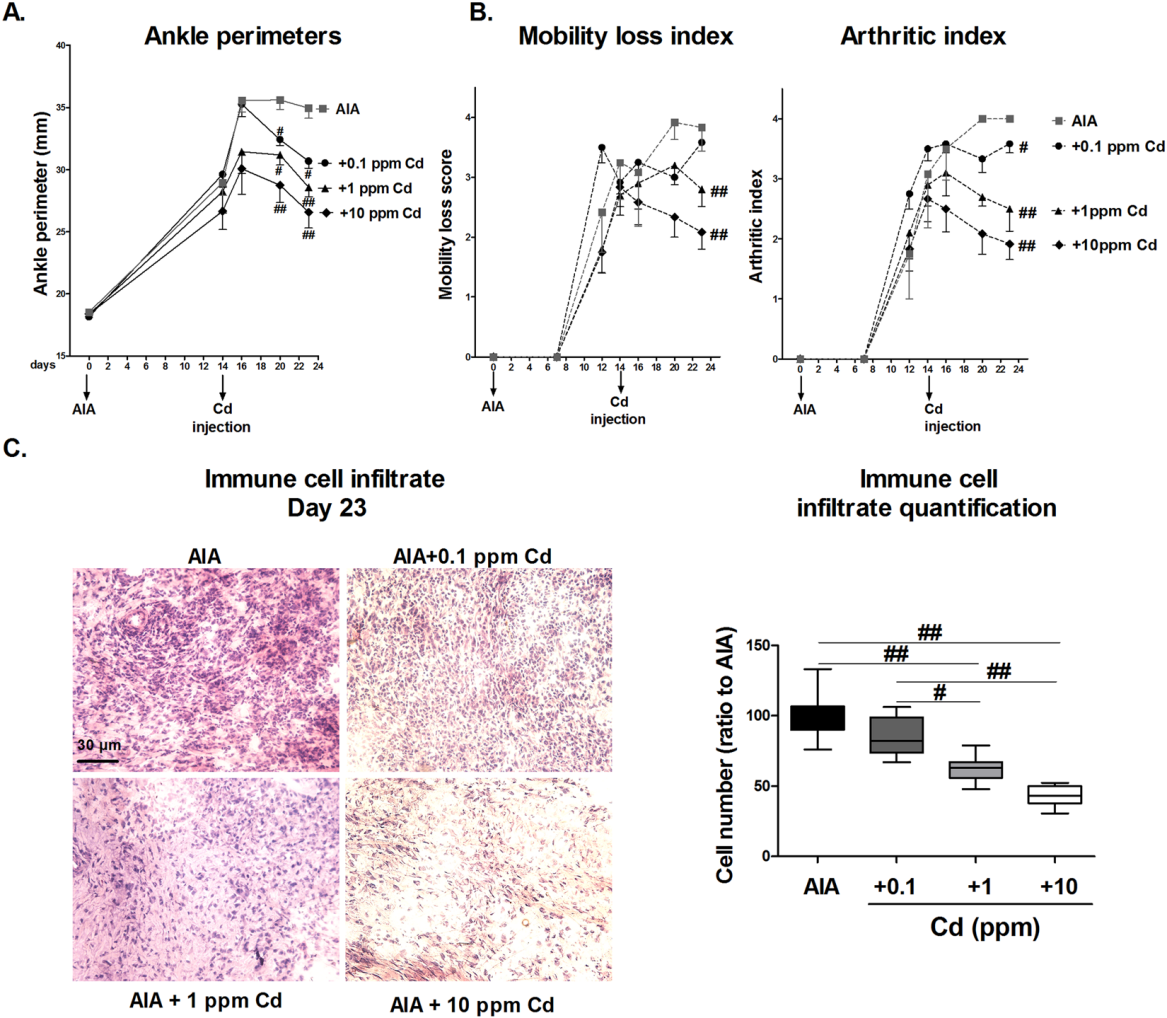
Cd intra-articular injection improves clinical scores and reduces inflammation in AIA rats. (**A**) Ankle perimeters of rat hind paws in function of time. Day 0: AIA induction; day 14: Cd intra-articular injection. (**B**) Clinical scores of AIA; Left panel: mobility loss index from 0 = normal use of paws to 4 = complete loss of paw support; right panel: arthritic index from 0 = no swelling or erythema to 4 = excess oedema with joint rigidity. (**C**) H&E staining and measurement of immune cell infiltrate plus synoviocytes on joint pannus layers. Data are the mean of two independent measures on 6 animals per group, excepting the AIA plus Cd-1 ppm group, counting 5 animals. Data are represented as mean ± SEM; ^#^p < 0.05, ^##^p < 0.01.

Ankle perimeters and clinical scores were used to measure disease activity. No difference in paw perimeters was observed between groups at day 0 (AIA induction). At day 23, paw perimeters of AIA non-treated rats had increased by 16.4 mm ± 2.9 mm in comparison to day 0. The delta change of perimeters between day 0 and day 23 was reduced in Cd-treated groups to 12.5 ± 2.2 mm, 10.2 ± 2.5 mm and 8.2 ± 4.3 mm in the 0.1, 1 and 10 ppm groups, respectively. A significant reduction (p < 0.01 for 10 ppm, p < 0.05 for 1 and 0.1 ppm) of ankle perimeters occurred after Cd-treatment, compared to control rats (Fig. 3A). Thus, the reduced swelling was directly proportional to the amount of injected Cd.

To extend these results, other clinical parameters were measured (Fig. 3B). Both the arthritic and the mobility-loss score had a reduction of 2 out of 4 points with Cd at 10 ppm and 1.5 out of 4 with Cd at 1 ppm (p < 0.01 in comparison to the control group). The decreased arthritic score indicated a reduction of swelling and redness and the decreased mobility-loss index an improved use of joints (Fig. 3B).

H&E staining showed a significant reduction of the cell accumulation in the pannus of Cd-treated rats, referring to the infiltrate immune cells and synoviocytes (Fig. 3C, p < 0.01). This resulted in a 50% reduction in the 10-ppm group (p < 0.01) and 35% in the 1 ppm treated group (p < 0.01), in comparison to the control.

**Figure 4:**
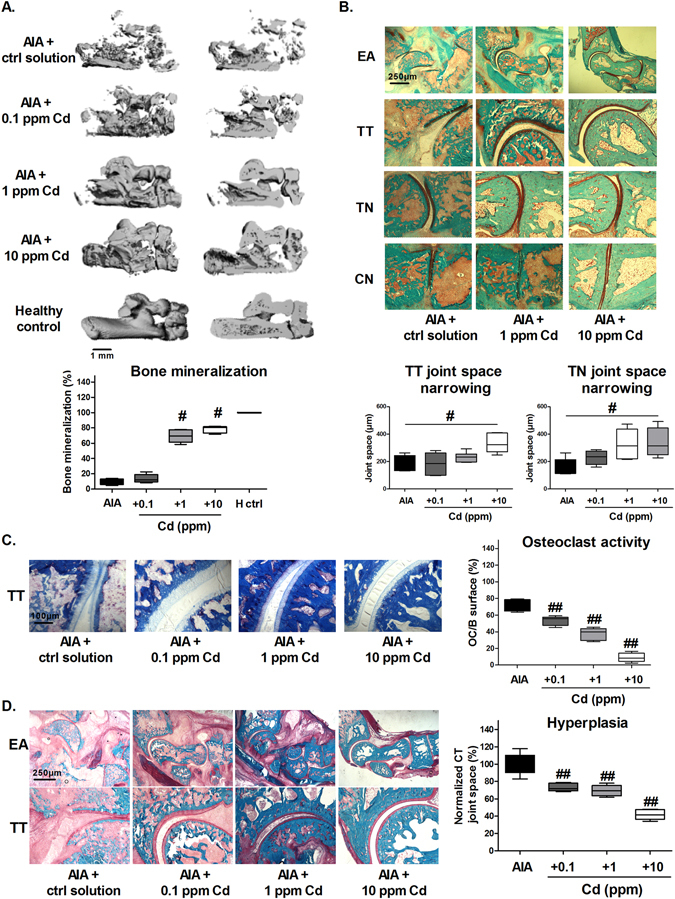
Cd intra-articular injection reduces inflammation-induced bone loss and cartilage destruction in AIA rats. (**A**) 3D-images reconstructed after micro-computed tomography analysis of hind ankle bones and quantification of bone mineralization of navicular bone (**B**). Safarin-O Cartilage staining and joint space narrowing quantification between talus and tibial or talus and navicular bones. Mineralized tissue in green, cartilage in red-orange. (**C**) TRAP staining in the TT space and quantification of osteoclast activity (**D**). Representative modified trichromatic Goldner staining on ankle sections: blue/green/turquoise: mineralized tissue; red periosteal layer and non-mineralized bone matrix; yellow/orange: bone marrow and other soft tissues; quantification of hyperplasia as calcaneo tibial joint space. EA: entire ankle; TT: talo-tibial; TN: talo-navicular; CN: cuneo-navicular. Data are the mean of two independent measures on 6 animals per group, excepting the AIA plus Cd-1 ppm group, counting 5 animals. Data are represented as mean ± SEM; ^#^p < 0.05, ^##^p < 0.01.

Although the reduction of inflammation was already of interest, control of bone loss remains the key target in arthritis^29^. To quantify the effect of Cd-induced reduction of inflammation on bone loss, ankles were scanned by micro-computed tomography (μ-CT) at necropsy (Fig. 4A) and bone moralization was quantified. The tri-dimensional reconstruction showed the conservation of bone quantity and quality with Cd at 1 and 10 ppm in the navicular, cuboid, talus, and calcaneus bones, resulting in a significant increase of bone mineralization (Fig. 4A, p < 0.05).

The effects of Cd-injection on cartilage integrity were observed by Safarin O-fast green staining and measured as joint space narrowing (Fig. 4B). The 10 ppm-Cd injection preserved cartilage (in red-orange) between the talo-tibial and talo-navicular spaces (p < 0.05) and abundant and well organized chondrocytes were observed. TRAP staining of the talo-tibial space was lower in the Cd treated groups, indicating a lower osteoclast activity in all cadmium conditions (Fig. 4C, p < 0.01). Modified Goldner trichromatic staining confirmed the increase in bone mineralized matrix and showed a reduced synovium hyperplasia in all the Cd treated groups, measured as calcaneo-tibial joint space (Fig. 4D, p < 0.01). These results provide strong arguments for the protective effects of intra-articular Cd-administration on joint inflammation and destruction.

### Cadmium spread in tissues and organs out of the joint is limited and not harmful

**Figure 5:**
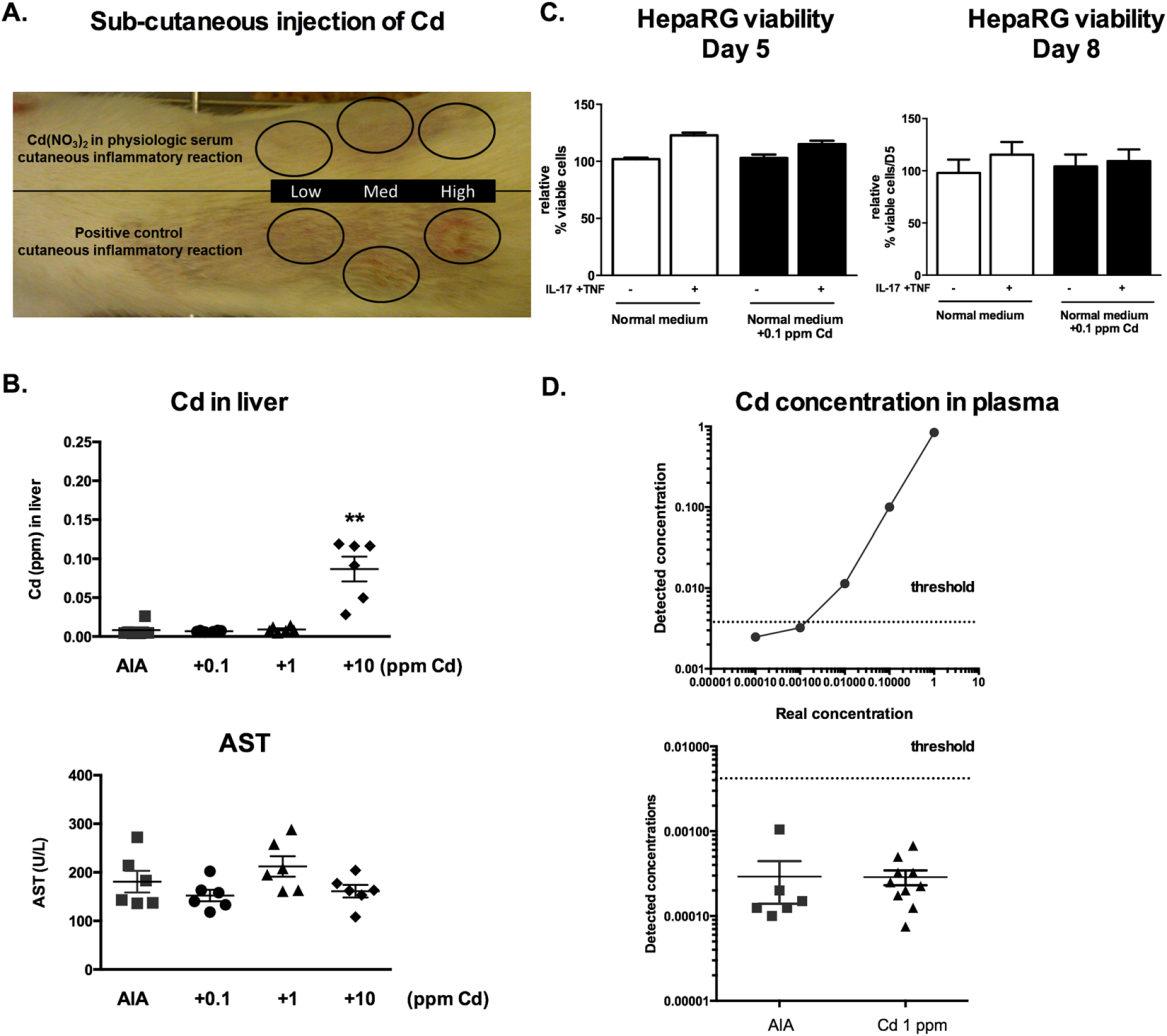
Cadmium spread in tissues and organs from the joint is limited and not harmful. (**A**) Subcutaneous injection of Cd and positive inflammatory control at increasing concentrations. (**B**) Measures of Cd and AST concentrations in liver tissue taken at necropsy on day 23. (**C**) Hepatocyte cell viability measured by neutral read assay after 5 and 8 days of exposure to Cd. (**D**) Plasma Cd concentration. On the upper panel known concentrations of Cd were measured to define the detection limit threshold. On the lower panel Cd concentration detected in plasma of 1 ppm treated rats. Dashed line represent detection limit threshold. Results represent independent measures on at least 5 animals per group. Data are represented as mean ± SEM; **p < 0.01.

Having demonstrated efficacy, we next looked at safety. At the local site, the sub cutaneous Cd-injection, (top Fig. 5A) in the back of a healthy age-matched Lewis rat, did not cause changes whereas a nitric acid injection used as positive control induced skin inflammation at the injection site (bottom Fig. 5A).

We next looked for a possible systemic effect. Although the pharmaco-kinetics of intra-articular Cd have not been described, acute Cd-exposure may have liver toxicity^30^, different from the effects of chronic exposure on kidney^31^. Since this study used a single injection, the effects of acute Cd-exposure were monitored by measuring Cd-concentration in rat liver. At necropsy, Cd was detectable only in liver biopsies of the 10 ppm treated rats (Fig. 5B; p < 0.01). The liver concentration was still three orders of magnitude lower than the 10 ppm injected in the joint (0.1 ppm found in liver). Levels of aspartate transaminases (AST), the most specific marker of liver damage for rats, remained unchanged between groups and similar to age-matched healthy control levels (AST mean: 246 U/L) (Fig. 5B).

To extrapolate the observations to the human situation, the effect of 0.1 ppm of Cd, as detected in the liver, was tested on a human hepatocyte cell line. At day 5 and 8 after Cd-exposure, in the presence or not of IL-17/TNF-α, no change in cell viability was observed (Fig. 5C).

To establish the ICP-MS detection limits, known concentrations of Cd were added to human plasma and Cd levels were measured. The Cd detection limit was as low as 0.01 ppm (Fig. 5D). Plasma from 1 ppm Cd-treated rat group were then analysed and no Cd could be detected in these samples.

Based on the results in the rat model, the 1 ppm-dose, corresponding to 50 ng of Cd, could be selected as providing the best risk/benefit. With this dose, the protective effects of Cd were observed in the joint and surrounding bone and cartilage, without systemic leakage and adverse effects outside.

## Discussion

The increasing need for an improvement in intra-articular treatment for synovial hyperplasia has not been overcome by current systemic therapies. In the present study, exposure to Cd had an anti-proliferative effect on isolated synoviocytes and RA synovial biopsies, associated with a reduced inflammation. Finally, this proof of concept study shows that intra-articular Cd-injection in the AIA rat joints could control inflammation, bone loss and joint destruction.

Cd accumulation in synoviocytes reduced cell viability, cell proliferation and IL-6 levels in supernatants. Annexin-V staining showed a higher sensitivity of OA synoviocytes exposed to Cd in comparison to RA. Moreover, OA synoviocytes were sensitive to the contact with PBMCs and their cell viability was slightly reduced in co-cultures. These results support the acquired resistant phenotype of RA synoviocytes^32^. Nevertheless, the association of Cd and IL-17/TNF combination highly induced cell death in RA synoviocytes. The reduced inflammation, associated with annexin-V staining indicated that cells undergo apoptosis, a cell death process characterized by the lack of inflammation, differently from other processes of cell death, such as pyroptosis^33^.

As described in our previous work, synoviocyte contact with PBMCs was sufficient to induce a large release of IL-6^27^. Cd treatment did not reduce PBMCs viability and their IL-6 secretion (data not shown), probably due to the down-regulation of Zn transporters on activated PBMCs^34^. Conversely, Cd reduced IL-6 production in synoviocytes cultured alone or in co-culture, implying a main effect of Cd on synoviocytes. Co-culture and biopsy experiments confirmed the modulatory effect of Cd through the pro-apoptotic pathway of synoviocytes, implying a reduced attraction of immune cells at the site of inflammation, in turn reducing the level of cell-cell interactions.

ZIP-8 and ZIP-14 importers are closely related and participate in the transport of Zn and Cd^35, 36^. Exposure to IL-17/TNF-α combination increased ZIP-8, but not ZIP-14 (data not shown) expression in synoviocytes. Furthermore, ZIP-8 and MT-1 increased expression in inflammatory synoviocytes was associated with an irreversible accumulation of Cd. Absorption of residual Cd in the ppb range continued after washing. This indicates the absence of Cd release, independently of the overexpression of the exporter ZnT1, which is triggered by the Cd-dependent MTF-1 activation^37, 38^. The lack of Cd export associated with ZnT1 over-expression did not clarify if ZnT1 is the best candidate for Cd export from synoviocytes, or if there is no export of Cd at all. Indeed, no direct measurement of Cd-export in mammalian cells is available and linked to one specific transporter. Mammalian ZnT have been considered as the best candidates for Cd export, but these transporters have an histidine pairing at the metal transport site that controls Zn over Cd selectivity^39^. Other transporters such as CFTR/ABC-C7 are not proven Cd-transporters and Cd-ligand complexes have a too low affinity to be exported by other transporters^40^. The lack of an identified candidate suggests that Cd-induced cellular damage could be a major contributor of Cd exit from the cell. In the context of this study, Cd linked to MTs was retained in synoviocytes, inducing cell death. Cell death may be the only way for Cd massive export from the cell. The high expression of ZIP-8 importer and MTs by inflammatory synoviocytes could imply a rapid re-uptake of the Cd released by injured cells by other synoviocytes *in situ*, avoiding systemic Cd spread. This would reduce systemic toxicity.

To extend the *in vitro* effects of low-dose Cd, an *in vivo* model of arthritis was used. The intra-articular Cd-injection was performed when arthritis was active, as would be the case in arthritic patients. The results showed a clear effect on inflammation and related destruction with increasing Cd-concentrations, always keeping exposure at sub-toxic doses (calculated from the rat body mass, per the WHO recommendation in humans). This is the first study where infinitesimal doses of Cd (total amount of Cd injected: 5, 50 or 500 ng) were used and still showed a positive effect. More than the control of inflammation, the effect on bone loss remains a critical issue in arthritis. Administration of Cd showed no negative effects on cartilage, with no obvious damage to chondrocytes and no joint space narrowing. The key finding is that Cd administration preserved bone from destruction. Cd main effect *in vitro* is thus the induction of synoviocyte apoptosis, further reducing inflammation. The direct effect of Cd on immune cells appears limited, in line with the absence of cell death in PBMCs. In the *in vivo* model, both hyperplasia and inflammation were reduced, leading to a reduction of damage to cartilage and juxta-articular bone.

Further investigation is needed on Cd-transporters and MT expression and their response to the inflammatory and Cd stimuli in chondrocytes and bone cells. MT-2 but not MT-1 was suggested as a regulator of chondrocyte apoptosis in OA pathogenesis and was reported to be overexpressed in damaged tissue in a rat model^41^, while ZIP-14 on chondrocytes contributes to bone formation through Zn import^42^.

Considering safety, the possible toxicity of acute administration of Cd was evaluated at the major accumulation sites of known systemic Cd toxicity, as Cd pharmaco-kinetics via intra-articular administration are unknown. Nevertheless, intra-articular exposure to Cd represents a local exposure, which could be less toxic than other types of exposure, e.g. inhalation. Sub-cutaneous Cd injections did not induce local inflammation. The major known site of accumulation during Cd acute exposure is the liver^30^. In the treated rats, Cd was detected in liver only at the high Cd-dose group (10 ppm), at a liver concentration of 0.1 ppm. Cd at this 0.1 ppm concentration did not alter the survival of a hepatocyte cell line. Transaminase levels in rats remained unchanged, suggesting no obvious liver cytotoxicity.

When transposing these results to the human situation, the proposed amount of 1 ppm of Cd would correspond to the exposure to the smoke of about 3 cigarettes^43^. For arthritis, the Cd administration would be a single or a few intra-articular injections and this could provide a good risk/benefit ratio, better than with the use of radio-active isotopes.

In conclusion, this proof of concept study provides the basis for the use of low dose Cd for intra-articular treatment of local inflammation. Further studies are needed to explore other modalities in humans such as the use of Cd-based quantum dots to limit diffusion^44^. The first indication could be the orphan disease PVNS, where the cancer-type proliferation of the synovium is difficult to control, even with surgery.

## Methods

### Biopsy and cell culture

Synoviocytes were grown from fresh synovial tissue samples aseptically isolated from RA and osteoarthritis (OA) patients’ joints. The RA patients fulfilled the American College of Rheumatology criteria for RA. All patients signed an informed consent form and the study was approved by the ethics committee of the hospitals of Lyon. All methods were performed in accordance with these guidelines and regulations.

The synovial tissue, minced in small pieces, was allowed to adhere to plastic plates and maintained in DMEM medium (Eurobio, Courtaboeuf, FR) supplemented with 10% FBS (Life Technologies by Thermo Fischer scientific, Grand Island, NY, USA), 2% Penicillin-Streptomycin, 1% L-glutamine and 1% Amphotericin B (all Eurobio) until cells reach 90% confluence. Synoviocytes were used between the fourth and ninth passages to ensure the cell specificity. Biopsies, RA and osteoarthritis (OA) synoviocytes and peripheral blood mononuclear cells (PBMCs) were cultured alone or in co-cultures and exposed to the combination of 50 ng/ml of IL-17A and 0.5 ng/ml of TNF-α or phytohemagglutinin 5 μg/ml (PHA) as already described^27^. HepaRG hepatocyte cell line was cultured as previously described^45^. The day after 0.1, 1 or 10 part per million (ppm, µg/ml) of Cd were added to the culture medium at different time intervals. The protocol was approved by the Ethics Committee of the Hospitals of Lyon for the protection of persons participating in biomedical research under the number AC-2010-11-64.

### Cd cell content and kinetics by ICP-MS

Cd concentration in synoviocytes (5 * 10^5^), medium, liver tissue, rat and human plasma was measured by Inductively-coupled-plasma mass spectrometry (ICP-MS) as described previously^11^ Synoviocytes (5 * 10^5^) were cultured with cytokines and metals being optionally added. Supernatants (2 ml) and cells were uptake at the end-point (day 14 for metal cocktail and day 5 for Cd only) to analyse metal fractionation constants K_D_. *K*_D_ between cells and medium was calculated as *K*_D_ = (Me/^70^Zn)_cells_/(Me/^70^Zn)_medium_. Values of *K*_D_ of ~1 indicate an isotopic equilibrium, whereas *K*_D_ > 1 indicates that exchange reactions are still on their way.

To analyse Cd kinetics and cell content, 2 ml of supernatant were collected at 6, 12, 24, 48 hours. Cells were than washed with PBS and fresh complete DMEM was substituted to the Cd-enriched medium, to study the possible exit of Cd from cells. Two ml of medium were collected immediately after the wash, at 60, 72, 96 and 120 hours, while cells were collected and counted at the endpoint (120 hours). All the samples were then mineralized with HNO_3_ 0.5 N plus H_2_O_2_ (15–20%) at 100 °C. Prior the analysis, the mineralized samples were re-dissolved in a 5% HNO_3_ solution in deionized water in an ultrasonic bath. Metal ions in the samples were then measured on a single collector ICP-MS platform ELEMENT 2 (Thermo Finnigan, Ringoes, NJ, USA) which allows metals to be measured at concentrations levels as low as 10^−12^ units, i.e. in the part per trillion (ppt) range. The detector receives an ion signal proportional to the metal concentration. The same analysis was performed on mineralized liver tissue and plasma of rats obtained after rat sacrifice.

### Gene expression of Zn transporters by quantitative real-time PCR

Synoviocytes (2.5 * 10^5^) were exposed or not to cytokines overnight followed or not by 6-hour Cd exposure. The expression of ZIP-8, MT-1F, MT-1M, MT-1X and ZnT1 were quantified as previously described^11^. After 6 hours of treatment, total RNA was extracted using the RNeasy Mini Kit (Qiagen®, Hilden, GE) and quantified with the Quant-it kit assay (Invitrogen™ by Thermo Fisher Scientific, Grand Island, NY, USA) following manufacturer’s instructions. cDNA was synthesized using the QuantiTect reverse transcription kit (Qiagen®) according to the manufacturer’s instructions. SYBR green-based real time qRT-PCRs were performed on the CFX96 Real-Time PCR Detection System (BioRad, Hercules, CA, USA) using the QuantiFast SYBR green kit and QuantiTect primers (Qiagen®). Cycle threshold values were normalized with respect to the endogenous control gene glyceraldehyde 3-phosphate dehydrogenase (GAPDH). The relative expression of the genes in treated cells versus control cells was determined using the comparative threshold cycle method as described by the manufacturer.

### Cell viability and IL-6 production

Synoviocytes were plated at a density of 10^4^ cells/cm^2^ in 96-well plates in normal cultures, at 7.5^4^ cells/cm^2^ in co-cultures with PBMCs were used in a ratio 1:5. After exposure or not to cytokines and Cd for 1, 5 and 8 days, cells were incubated for 150 min with neutral red dye (80 µg/ml 0.33%; Sigma-Aldrich, St. Louis, MO, USA) at pH 6.5 in serum free DMEM. Confluent HepaRG cells viability after Cd exposure was also assessed by neutral red assay with the same protocol.

Biopsies were minced in small pieces of similar size and put in 24 well plates in normal DMEM medium (Eurobio, Courtaboeuf, FR). Supernatants were collected at day 0 for IL-6 production normalization and biopsies were treated afterwards with cytokines and Cd in triplicates. Cell proliferation was measured by the area occupied by the cells on the plate after 5 days using ImageJ™ software. Supernatants were collected at day 2, 5 and 8 after Cd exposure.

IL-6 production was quantified in supernatants by ELISA (R&D system, San Diego, CA, USA).

### Annexin V staining

Synoviocytes (10^4^ cells/cm^2^), after exposure or not to cytokines and Cd were incubated with Annexin V-FITC (2 µg/ml). Fluorescence was measured after 5 days. Images (20x-Nikon™ camera on an Axiovert200 microscope) were analyzed on CellProfiler™. The stained area was calculated with a ratio on the total cell area.

### Induction of adjuvant induced arthritis (AIA)

AIA was induced in twenty-four 6-weeks old female Lewis rats (Charles River Laboratories, L’Arbresle, France) as described^28^, in accordance with the legislation of the European Community and approved by the Ethical Committee for Animal Experiments of Saint Etienne University (authorization #CU14N14). At day 14, during the arthritis acute phase, 50 µl of Cd(NO_3_)_2_ 5% diluted in physiological serum (0, 0.1, 1 and 10 ppm) were injected into right and left hind ankles. This means that 5, 50 and 500 ng of Cd were injected in rat ankles, for each group respectively. At least five animals were used for each group.

Ankle perimeters, articular index and loss of mobility index were measured at different time-points (day 0, 12, 14, 16, 20, 23) as already described^28^.

### Histological examination and tri-dimensional tomography imaging

Animals were sacrificed at the end of standard acute phase in this model (day 23). Right ankles were collected at necropsy. Sections were stained with H&E and the immune infiltrate was measured by Safranin O-fast green staining was used to identify cartilage integrity and immune infiltrate; Modified Goldner trichrome staining (picric acid, fuchsin, phospholimbidic acid and light green) was used to identify bone and synovium morphology; Tartrate resistant acid phosphate (TRAP) staining was used as osteoclast activity (in red) and bone tissue (in blue). Joint space narrowing was measured between talus and tibial or talus and navicular bones. Ankles were scanned by micro-computed tomography (µ-CT; viva-CT40, Scanco, Brütisellen, Switzerland) and bone mineralization was measured as already described^28^. X-rays beam features included: energy: 55 kVp, intensity: 145 µA, diameter: 25.6 mm, voxel size: 12.500 µm^3^, resolution: high, as previously described^28^. 3D images were constructed with the following segmentation parameters: Gauss sigma: 3.4, Gauss support: 9, Lower threshold: 316, Higher threshold: 1000).

### Statistical analysis

Data are expressed as the mean ± standard error of the mean (SEM). Statistical significance was determined by GraphPad Prism™. A two-way ANOVA test, followed by multi-parametric analysis, was used for exposure to both Cd and cytokines. A non-parametric Mann-Whitney paired test was used for single exposure, with pairing between same patient cells. p-values inferior or equal to 0.05 were considered statistically significant.
